# Top five ethical lessons of COVID-19 that the world must learn

**DOI:** 10.12688/wellcomeopenres.16568.1

**Published:** 2021-01-29

**Authors:** Maxwell J. Smith, Aasim Ahmad, Thalia Arawi, Angus Dawson, Ezekiel J. Emanuel, T. Garani-Papadatos, Prakash Ghimire, Zubairu Iliyasu, Ruipeng Lei, Ignacio Mastroleo, Roli Mathur, Joseph Okeibunor, Michael Parker, Carla Saenz, Beatriz Thomé, Ross E.G. Upshur, Teck Chuan Voo

**Affiliations:** 1 University of Western Ontario, London, Ontario, Canada; 2 The Kidney Centre Post Graduate Training Institute, Karachi, Pakistan; 3 American University of Beirut, Beirut, Lebanon; 4 University of Sydney, Sydney, New South Wales, Australia; 5 University of Pennsylvania, Philadelphia, Pennsylvania, USA; 6 University of West Attica, Athens, Greece; 7 Tribhuvan University, Kathmandu, Nepal; 8 Bayero University Kano, Kano, Nigeria; 9 Huazhong University of Science and Technology, Wuhan, China; 10 National Scientific and Technical Research Council, Buenos Aires, Argentina; 11 National Center for Disease Informatics and Research (ICMR-NCDIR), Bengaluru, India; 12 University of Nigeria Nsukka, Nsukka, Nigeria; 13 University of Oxford, Oxford, UK; 14 Pan American Health Organization, Washington, DC, USA; 15 Universidade Federal de São Paulo, São Paulo, Brazil; 16 University of Toronto, Toronto, Ontario, Canada; 17 National University of Singapore, Singapore, Singapore

**Keywords:** COVID-19, Ethics

## Abstract

As the world reflects upon one year since the first cases of coronavirus disease 2019 (COVID-19) and prepare for and experience surges in cases, it is important to identify the most crucial ethical issues that might lie ahead so that countries are able to plan accordingly. Some ethical issues are rather obvious to predict, such as the ethical issues surrounding the use of immunity certificates, contact tracing, and the fair allocation of vaccines globally. Yet, the most significant ethical challenge that the world must address in the next year and beyond is to ensure that we learn the ethical lessons of the first year of this pandemic. Learning from our collective experiences thus far constitutes our greatest moral obligation. Appreciating that decision-making in the context of a pandemic is constrained by unprecedented complexity and uncertainty, beginning in June 2020, an international group of 17 experts in bioethics spanning 15 countries (including low-, middle-, and high-income countries) met virtually to identify what we considered to be the most significant ethical challenges and accompanying lessons faced thus far in the COVID-19 pandemic. Once collected, the group met over the course of several virtual meetings to identify challenges and lessons that are analytically distinct in order to identify common ethical themes under which different challenges and lessons could be grouped. The result, described in this paper, is what this expert group consider to be the top five ethical lessons from the initial experience with COVID-19 that must be learned.

As many countries reflect upon one year since the first cases of coronavirus disease 2019 (COVID-19) and prepare for and experience surges in cases, it is important to identify the most crucial ethical issues that might lie ahead so that countries are able to plan accordingly. Some ethical issues are rather obvious to predict, such as the ethical issues surrounding the use of immunity certificates ^1^, contact tracing ^2^, and the fair allocation of vaccines ^3^. Yet, the most significant ethical challenge that the world must address is ensuring that we learn the ethical lessons from earlier waves of this pandemic. Learning from our collective experiences thus far constitutes our greatest moral obligation, as failing to learn from our prior experiences and prevent the ethical shortcomings of earlier waves of the pandemic would represent a transgression of the ethical obligation to use available knowledge, evidence, and experience to protect and promote public health ^4^. Appreciating that decision-making in the context of a pandemic is constrained by unprecedented complexity and uncertainty, beginning in June 2020, an international group of 17 experts in bioethics spanning 15 countries (including low-, middle-, and high-income countries) met virtually to identify what we considered to be the most significant ethical challenges and accompanying lessons faced thus far in the COVID-19 pandemic. Once collected, the group met over the course of several virtual meetings to identify challenges and lessons that are analytically distinct in order to identify common ethical themes under which different challenges and lessons could be grouped. The result, described below, is what this expert group consider to be the top five ethical lessons from the initial experience with COVID-19 that must be learned.

## 1. We must learn to prepare adequately based on previous experience

The primary obligation of governments is to protect their populations from threats to their freedom and well-being. Yet, despite the laudable and often undervalued efforts of public health and emergency preparedness experts, we have collectively failed to make the necessary preparations for pandemics despite knowledge about the risks. We have also been slow to learn from the experiences of other countries who were affected earlier in this pandemic. Adequate preparation is an ethical obligation because it is, all other things being equal, better to prevent harms from occurring rather than only respond once they emerge. Consequently, where we know harms will occur, we have an ethical obligation to mitigate them. Features of public health systems such as adequate surveillance, stockpiles of personal protective equipment, testing, contact tracing, the identification of at-risk populations, and other non-pharmaceutical interventions are crucial to the prevention and mitigation of harms, and must be strengthened, particularly in the absence of a widely available vaccine and the existence of only limited treatments. Given the world’s experience with COVID-19, an ethical obligation exists to learn from previous experience and aggressively seek to prevent these predictable harms from occurring in subsequent waves.

## 2. We must learn to better articulate and prioritize overarching goals

The policies and measures implemented in response to the pandemic should align with a set of clear and justified overarching goals. Such goals inevitably engage with important and often conflicting ethical considerations. In the COVID-19 pandemic, there has been a lack of clarity as to whether the overarching goal has been to flatten the curve, protect the acute health care system, reduce morbidity and mortality, protect the health of disadvantaged communities, protect those most vulnerable to infection, minimize extreme poverty, mitigate economic loss, or some combination of the above. Even if these were all possible goals for the pandemic response, more needs to be done to understand which goals should be prioritized over others, in what contexts, and at what point in the response, in addition to what justifies any proposed ranking.

The ethical case for requiring clear and justified overarching goals is that they are key to setting ethical and fair priorities for the distribution of vaccines, therapeutics, diagnostics, and other human and economic resources. Where resources are scarce, choices will have to be made about how they are allocated. One option is to distribute goods randomly, so that all have an equal chance of any benefit. However, many people believe that this is unethical, and that we have good grounds for choosing to distribute unequally taking into account other morally relevant features such as reducing mortality and morbidity directly and indirectly caused by the pandemic, reducing the social and economic consequences of the pandemic with particular emphasis on reducing health inequities, mitigating poverty, and ensuring continuity of important goods like education, and returning society to full functionality.

## 3. We must learn to work collaboratively

The most important aspect of a pandemic such as COVID-19 is that it is a global problem and therefore requires a global response. Even a focus on achieving what is best in terms of national self-interest requires solidarity across nations and between peoples to be most effective. The best protection for every country is to act together. We think it is therefore regrettable and unjust that some countries have chosen this moment to withdraw global social capital, support, influence, and funding. It is estimated that $3.4 billion (USD) a year is needed to fund the “global functions” of WHO pandemic preparedness, but funds have fallen woefully short of this target ^5^.

Other examples of failures to work together for the benefit of all include the lack of genuine international collaboration for surveillance and effective response as envisioned by the International Health Regulations ^6^, including early institution of mitigation measures, as well as the lack of collaboration within and between the countries in controlling access to personal protective equipment (PPE), diagnostic test kits, and reagents. Leaving the market to distribute essential goods during a global health emergency results in increased injustice because of the unequal access to available equipment and trained human resources.

## 4. We must learn to protect the most vulnerable individuals and populations

The pandemic and every pandemic policy will impact groups in a population unequally, and prior disadvantage is likely to be exacerbated. While it may often be difficult to anticipate the unforeseen consequences of prolonged public health and social measures on disadvantaged populations, we should acknowledge that prior disadvantage is likely to be exacerbated and plan for it. Support and resources must be distributed unequally to bring about greater equity. For example, poor preparedness for a pandemic like COVID-19 and the subsequent response imposed new norms of containment, physical distancing, and confinement orders without adequate financial support and other follow-up measures to mitigate their risks and harms. Consequently, about 100 million people are likely to be thrown into extreme poverty ^7^. In low- and middle-income countries, millions of low-paid daily wagers and migrant workers suddenly lost their jobs and were rendered homeless, with little access to food, clean water, and shelter ^8, 9^. There has been a failure to tackle the disproportionately negative impact this pandemic has had on certain communities like those living in slums or overcrowded places such as dormitories, prisons, refugee camps, and residential care facilities. Those living in these settings not only faced a greater risk of infection because of an inability to physically distance, but also faced the additional burden of stigma and discrimination because of their unwarranted association with increased transmission risks. Violence against women and the treatment of children and transgender people also adds to these layers of vulnerability ^10^.

The pandemic also adversely affected older adults or those with existing co-morbidities who missed their clinical appointments and follow-up care for potentially life-threatening chronic ailments. The lack of physical ‘family presence’ has also been devastating for dementia patients, among others, who rely on in-person family support ^11^. Additionally, millions of children not only missed out on routine immunizations and important healthcare interventions, faced food insecurity, and missed out on face-to-face education, playing with friends, and participating in physical activities ^12, 13^.

## 5. We must learn to improve communication

Conducting rigorous, expedited research without losing sight of scientific and ethical standards and appropriate dissemination of research results is essential in the context of a public health emergency so that individuals and communities can effectively protect themselves and navigate the ‘infodemic’ of false and misleading information ^14^. Yet, pre-prints of non-refereed studies have increased drastically with a large number of retractions ^15^. Some therapies touted in retracted studies have been the focus of ‘panic prescribing’, which in some cases has led to harm to patients ^16^. To learn from these experiences, two fundamental lessons about research and communication should be understood.

First, in general, evidence-based communication and a research-literate culture promotes societies that are more resilient to ideology and misinformation, whether in deciding about policies of mask wearing, resource allocation, reopening the economy, or use of unproven interventions ^17^. Second, because good decision-making is based on accurate information, developing research skills is an investment for global society’s well-being and development, not a mere expense ^18^. Hence, carrying out research during pandemic times is at the same time a moral obligation of all countries ^19^. Politicians also need to take responsibly for their actions and not indulge in inappropriate involvement in issues of clinical expertise. A key part of any pandemic response is enabling trusted experts to provide clear and transparent translation of scientific findings, including the limitations of those findings, for the public.

## Conclusion

The five ethical lessons identified above are clearly not exhaustive of all ethical lessons or challenges experienced during this pandemic. They were identified by a group of bioethicists, physicians, and public health practitioners from around the world and reflect our collective experience and judgements and should be interpreted as such. Consequently, we have little doubt that serious ethical lessons—perhaps ones even more significant than those we identified—may have been neglected. Nevertheless, we believe that learning these lessons constitutes our greatest moral obligation as this pandemic continues to evolve.

## Disclaimer

The opinions in this open letter are those of the authors and do not necessarily reflect the views of the authors’ institutions.

## Data availability

### Underlying data

No data are associated with article.
