# Supplementary material for: Top five ethical lessons of COVID-19 that the world must learn
Source: Wellcome Open Res. 2021 Jan 29;6:17. [Version 1] doi: 10.12688/wellcomeopenres.16568.1 (PMC7919608; doi:10.12688/wellcomeopenres.16568.1)
Supplement: Peer review history

# Peer review: Reviewer response for version 1

**DOI:** 10.21956/wellcomeopenres.18258.r42411

**Published:** 2021-02-26

**Authors:** Gamal Serour

**Affiliations:** 1 Department of Obstetrics and Gynaecology, Al Azhar University, Cairo, Egypt

The global spread of COVID-19 since the beginning of the last year has struck the world not only with health, educational, economic, and social problems, but also created serious ethical concerns.

 An International well-qualified group of 17 experts bioethics spanning 15 countries including low, middle, and high income countries met virtually and identified in their opinion the most significant ethical challenges and companying lessons based the world in its combat of COVID-19 pandemic.

 Five top lessons were identified by the group in this regard and indeed they highlighted important ethical lessons raised when the whole world developed and developing suffer from the unprecedented spread of the COVID-19 pandemic.

- **The first lesson** identified was that the world must learn to prepare adequately to face similar unexpected pandemics to perform better than we did during the current pandemic.
- **The second lesson** was that we must learn the better articulate and priorities overarching goals. No doubt during the pandemic several healthcare, educational, economic, social and mental issues were faced. We globally did not prioritise the overarching goals and kept on complete lockdown, partial lockdown, no lockdown and shifting from one lockdown to another. This resulted in serious impairments of service provided in sexual reproductive health, including contraception, gender based violence, educational services  and economic crisis.
- **The third lesson** highlighted how important it is for the world to learn to work collaboratively whether at the governmental levels, UN organizations, Pharmaceutical Industries, private sectors, and civil society organizations.
      
- **The fourth lesson** was how we should in such circumstances learn to identify and protect the most vulnerable individuals and populations as the elderly, the diseased women and children and not forget specifically health care providers who are almost always at the forefront in saving the lives of our people. The world lost many of the health care providers and elderly because they were not timely provided with the necessary protective equipment and the care they needed.
- **The fifth lesson** which I believe is an extremely important lesson is that we all in different parts of the world developed, or developing countries must learn to improve our communications to combat better such similar pandemics. The positive achievements, policies, researches and treatment must be communicated to all others immediately for the benefit of the whole of humanity. We are living in a small village and what happens in the far east affects those in the far remote areas whether Africa, Europe, North America or South America and vice versa.

No doubt these are important ethical lessons identified by this expert group and they admit that these five lessons are not exhausted of all ethical lessons or challenges, experienced during this pandemic and I could not agree more.

Does the article adequately reference differing views and opinions?

Yes

Are all factual statements correct, and are statements and arguments made adequately supported by citations?

Yes

Is the Open Letter written in accessible language?

Yes

Where applicable, are recommendations and next steps explained clearly for others to follow?

Yes

Is the rationale for the Open Letter provided in sufficient detail?

Yes

Reviewer Expertise:

Bioethics, Infertility, SRHR, Assisted reproductive technology.

I confirm that I have read this submission and believe that I have an appropriate level of expertise to confirm that it is of an acceptable scientific standard.

# Peer review: Reviewer response for version 1

**DOI:** 10.21956/wellcomeopenres.18258.r42415

**Published:** 2021-02-15

**Authors:** Gustavo Ortiz-Millan

**Affiliations:** 1 Institute for Philosophical Research, National Autonomous University of Mexico, Mexico City, Mexico

It is true that we must learn to prepare adequately based on previous experience. It is also true that we have also been slow to learn from the experiences of other countries that were affected earlier in this pandemic. However, we have also been slow to learn from previous pandemics. The rate at which epidemics have appeared in the last 30 or 40 years has been greater than ever before. When the authors talk about the obligation to prepare adequately, they focus on “features of public health systems such as adequate surveillance, stockpiles of personal protective equipment, testing, contact tracing, the identification of at-risk populations, and other non-pharmaceutical interventions.” I wonder whether by “adequate surveillance” they are also thinking about surveillance of zoonotic outbreaks.

 There is something that we have not learned despite having gone through other epidemics in recent times: the origin of this pandemic is in the way we relate to animals and exploit them. Similar epidemics that we have seen in recent decades have their origin in zoonotic outbreaks, that is, they were transmitted from animals to humans. According to the CDC, three out of every four emerging infectious diseases in people come from animals. ^1^ Recent pandemics such as HIV/AIDS, ^2^ Ebola, ^3^ MERS, ^4^ H7N9, ^5^ H1N1, ^5^ H5N1, ^6^ and SARS (now known as SARS-CoV-1), ^7^ among others have a zoonotic origin. Many (if not all) of them arose in contexts of exploitation of animals (wild or domestic), such as the trade—legal or illegal—of wildlife species, or the production of meat in intensive systems in factory farms. Some even took place in “wet markets” in China or Southeast Asia—such as H5N1, H7N9 and the SARS epidemics. ^8^ Wildlife trade and factory farms have grown in recent years, oftentimes in contexts of little or no regulation or supervision by the health and environmental authorities of the countries where these activities take place, but sometimes in contexts of illegality. These types of activities have increased the likelihood of outbreaks of emerging infectious diseases.

 ​​​​​​Some epidemic diseases also have an environmental origin, related to the invasion of habitats and to the close contact that the natural reservoir fauna is forced to have with domestic animals and, ultimately, with humans. They also have to do with biodiversity loss and the increasing prevalence of certain pathogens in reservoir populations. If biodiversity does not deteriorate, reservoir populations are diluted among other species, and the risk of pathogens transmission is reduced.

 ​​​​​​Apparently, we have not learned much from previous pandemics, but if there is a lesson to be learned from the current one, is that if we do not modify our relationship with animals (and nature in general), it is very likely that we will see the emergence of other zoonotic pandemics in the near future.

Does the article adequately reference differing views and opinions?

Yes

Are all factual statements correct, and are statements and arguments made adequately supported by citations?

Yes

Is the Open Letter written in accessible language?

Yes

Where applicable, are recommendations and next steps explained clearly for others to follow?

Yes

Is the rationale for the Open Letter provided in sufficient detail?

Yes

Reviewer Expertise:

Bioethics

I confirm that I have read this submission and believe that I have an appropriate level of expertise to confirm that it is of an acceptable scientific standard.

## References

[1] : Zoonotic diseases.2017; Reference source

[2] : AIDS as a zoonosis: scientific and public health implications. *Science*.2000;287(5453) : 10.1126/science.287.5453.607 607-14 10.1126/science.287.5453.607 10649986

[3] : Mapping the zoonotic niche of Ebola virus disease in Africa. *Elife*.2014;3: 10.7554/eLife.04395 e04395 10.7554/eLife.04395 25201877PMC4166725

[4] : MERS Coronavirus: An Emerging Zoonotic Virus. *Viruses*.2019;11(7) : 10.3390/v11070663 10.3390/v11070663 PMC666968031331035

[5] : Influenza (Avian and other zoonotic).2018; Reference source

[6] : Zoonotic transmission of avian influenza virus (H5N1), Egypt, 2006-2009. *Emerg Infect Dis*.2010;16(7) : 10.3201/eid1607.091695 1101-7 10.3201/eid1607.091695 20587181PMC3321902

[7] : Discovery of a rich gene pool of bat SARS-related coronaviruses provides new insights into the origin of SARS coronavirus. *PLoS Pathog*.2017;13(11) : 10.1371/journal.ppat.1006698 e1006698 10.1371/journal.ppat.1006698 29190287PMC5708621

[8] : Seven Modern Plagues and How We are Causing Them. *Island Press*.2014;
